# Breathing Pattern Response after 6 Weeks of Inspiratory Muscle Training during Exercise

**DOI:** 10.3390/arm92010008

**Published:** 2024-01-17

**Authors:** Eduardo Salazar-Martínez

**Affiliations:** Centro de Estudios Universitarios Cardenal Spínola CEU, 41930 Sevilla, Spain; esalazar@ceu.es

**Keywords:** breathing pattern, ventilation, cycling, inspiratory muscle training

## Abstract

**Highlights:**

**What are the main findings?**

- Inspiratory muscle training (IMT) improves the strength and performance of respiratory muscles.
- Improvements in respiratory performance do not have a relationship with modifications in the breathing pattern.

**What is the implication of the main finding?**

- IMT can be an effective intervention for enhancing the respiratory response.
- IMT does not alter the central response to respiratory control in healthy subjects.

**Abstract:**

(1) Background: The breathing pattern is defined as the relationship between the tidal volume (VT) and breathing frequency (BF) for a given VE. The aim of this study was to evaluate whether inspiratory muscle training influenced the response of the breathing pattern during an incremental effort in amateur cyclists. (2) Methods: Eighteen amateur cyclists completed an incremental test to exhaustion, and a gas analysis on a cycle ergometer and spirometry were conducted. Cyclists were randomly assigned to two groups (IMTG = 9; CON = 9). The IMTG completed 6 weeks of inspiratory muscle training (IMT) using a PowerBreathe K3^®^ device at 50% of the maximum inspiratory pressure (Pimax). The workload was adjusted weekly. The CON did not carry out any inspiratory training during the experimental period. After the 6-week intervention, the cyclists repeated the incremental exercise test, and the gas analysis and spirometry were conducted. The response of the breathing pattern was evaluated during the incremental exercise test. (3) Results: The Pimax increased in the IMTG (*p* < 0.05; d = 3.1; +19.62%). Variables related to the breathing pattern response showed no differences between groups after the intervention (EXPvsCON; *p* > 0.05). Likewise, no differences in breathing pattern were found in the IMTG after training (PREvsPOST; *p* > 0.05). (4) Conclusions: IMT improved the strength of inspiratory muscles and sport performance in amateur cyclists. These changes were not attributed to alterations in the response of the breathing pattern.

## 1. Introduction

Breathing is an essential function to keeping the body homeostatic [1]. The body’s ability to obtain oxygen and remove carbon dioxide relies on the effectiveness of the respiratory muscles. These muscles, specifically the diaphragm and intercostal muscles, are fundamental for the respiration and gas exchange processes. During exercise, breathing is related to exercise intensity, increasing the volume of gas inhaled–exhaled and breathing frequency as much as the increases in exercise demands. The breathing pattern is defined as the relationship between the tidal volume (Vt) and breathing frequency (BF) for a given ventilation (VE) [2]. In addition, VE can be decomposed in (a) central inspiratory activity (driving) expressed as the relationship between Vt and inspiratory time (Vt/Ti) and (b) the inspiration–expiration alternation (timing) expressed by the relationship between inspiratory time (Ti) and the total duration of the breathing cycle (Ti/Ttot) [3,4]. These variables have been commonly used to evaluate the breathing pattern in humans, ranging from a mechanical perspective (VT, BF) to the central nervous system control (VT/Ti; Ti/Ttot).

There are different stimuli that can modify the breathing pattern response. Among them are (a) hypoxia, (b) CO2 rebreathing, and (c) exercise. Concerning exercise, the breathing pattern has been investigated as a possible adaptation to exercise in athletes. For example, Lucía et al. [5] described the breathing pattern response in cyclists. They found that the breathing patterns of two cyclist groups of different levels (amateur vs. professional) differed mainly in the professional cyclists; VE increased in this group at any exercise intensity as a result of increases in both VT and BF. However, endurance training did not change the breathing pattern response during a competitive season in professional road cyclists [6]. Similarly, the breathing pattern of world-class cyclists did not change over three competitive seasons in spite of changes in cycling performance [7]. In soccer players, a 6-week detraining period did not change the BF and Ti/Ttot responses [8]. In this regard, it could be established that exercise has a residual effect on breathing patterns in highly trained athletes. Naranjo et al. [9] proposed a nomogram for assessing the breathing pattern response in athletes. Based on this analysis, BF and VT show an exponential relationship independent of the protocol used during the exercise test and regardless of how well trained the subject is.

Inspiratory muscle training (IMT) has been shown as an effective intervention for improving the respiratory muscle strength and exercise performance in different situations. A commonly used method for quantifying respiratory muscle strength is the measurement of maximal inspiratory (Pimax) and expiratory (Pemax) mouth pressures [10]. Two different IMT protocols (strength and endurance IMT) were effective in improving the Pimax and exercise performance in cross-country skiers [11]. The mechanisms proposed to explain the improvements reported after IMT are (a) hypertrophy of the diaphragm [12], (b) an increase in blood flow to the locomotor muscles [13], (c) a reduction in subjective perception of fatigue and dyspnea ratings [12], and (d) a greater mechanical efficiency [14].

In the sport field, respiratory performance has been shown to be an effective intervention for increasing overall performances in different sport disciplines [15,16,17]. However, from our knowledge, there are no previous studies that have investigated the relationship between inspiratory muscle performance and changes in breathing pattern. Understanding the interplay between inspiratory muscle performance and the breathing pattern is important in numerous contexts, from pulmonary health to sport performance. Evaluating whether the improvements after IMT are related to adjustments or changes in respiratory mechanics could provide greater insight into the contexts or populations where this methodology should be applied. Therefore, this study aimed to evaluate the influence of 6 weeks of IMT on the breathing patterns of amateur cyclists. We hypothesized that IMT could modify the response of the breathing pattern.

## 2. Materials and Methods

### 2.1. Subjects

Eighteen physically active and healthy participants [*n* = 9 male (23.44 ± 2.7 years; 180.22 ± 3.5 cm; 78.2 ± 5.5 kg; 48.39 ± 7.28 mL·kg^−1^·min^−1^); *n* = 9 female (24.27 ± 3.24 years; 166.35 ± 4.1 cm; 62.32.2 ± 8.47 kg; 39.15 ± 5.57 mL·kg^−1^·min^−1^)] were selected for the study. The inclusion criteria were (1) cycling at least 3 times per week, (2) healthy status, and (3) non-smoker. Before starting the study, written informed consent was obtained from each participant in accordance with the Declaration of Helsinki. The study was approved by the Institutional Review Board of the Department of Sport Science, University Innsbruck (Certificate of good standing 50_2015; 6 October 2015).

### 2.2. Design

Participants were randomly assigned to either an inspiratory muscle training group (IMTG; *n* = 9; 5 males and 4 females) or a control group (CON; *n* = 9; 4 males and 5 females). The IMTG performed 2 training sessions per day, 5 days per week during a period of 6 weeks. Each participant completed 30 inspiratory breaths against a restricted breathing flow device (PowerBreathe^®^, K3, Southampton, UK) at 50% of their individual Pimax. The inspiratory training load was adjusted weekly at 50% of their individual Pimax. Every training session was performed under expert supervision in the Sport Physiology Lab (University of Innsbruck) to ensure the adherence of the participants during the intervention period. The CON did not carry out any inspiratory training during the experimental period. Participants were advised not to change normal physical training habits during the experimental period.

### 2.3. Testing

Before (pre) and after (post) the experimental period, participants performed a spirometry (Schiller SP-1^®^, Baar, Switzerland) to assess the forced vital capacity (FVC), forced expiratory volume during the first second (FEV1), the ratio between the forced expiratory capacity during the first second and vital capacity (FEV1/VC), the peak expiratory flow (PEF), and the peak inspiratory flow (PIF) (Table 1). The best attempt out of three tests was included in the analysis. Maximal peak inspiratory mouth pressure (Pimax) was determined with a portable device (PowerBreathe^®^, K3, Southampton, UK). During the Pimax test, participants had to inspire as fast as possible from a residual volume after a maximal expiration. Pimax was measured weekly using the same testing protocol.

In addition, participants performed incremental exercise tests until exhaustion. During the tests, the oxygen uptake (VO_2_), carbon dioxide output (VCO_2_), respiratory exchange ratio (RER), ventilation (VE), breathing frequency (BF), tidal volume (VT), driving (VT/Ti), and timing (Ti/Ttot) were measured breath by breath with a gas analyzer (Jaeger OxygenTM^®^, Hoechberg, Germany). The system was calibrated prior to each test with gas mixtures of known concentrations. Tests were carried out on a cycle ergometer (RBM Cyclus 2^®^, Leipzig, Germany) under the same conditions, at the same time, and by the same researchers. After 4 min of warming up, participants started the test at 50 W, and then the load was increased by 25 W each minute until volitional exhaustion. The achievement of maximum oxygen uptake (VO_2max_) was accepted when a plateau was found in the relationship between VO_2_ and power output or when three of the four criteria for the VO_2max_ were obtained [18]. Participants were advised to avoid exhausting exercise 1 day before the tests and to take any ergogenic aids (e.g., caffeine).

### 2.4. Statistical Analysis

The normal distribution of the data was checked using the Shapiro–Wilk test. The homogeneity of variance was evaluated using Levene’s test. To compare the values obtained for each variable during the test, a paired sample *t*-test (PREvsPOST) and independent sample *t*-test (IMTGvsCON) were applied. The level of significance was set at *p* < 0.05 for each statistical analysis.

## 3. Results

**Figure 1:**
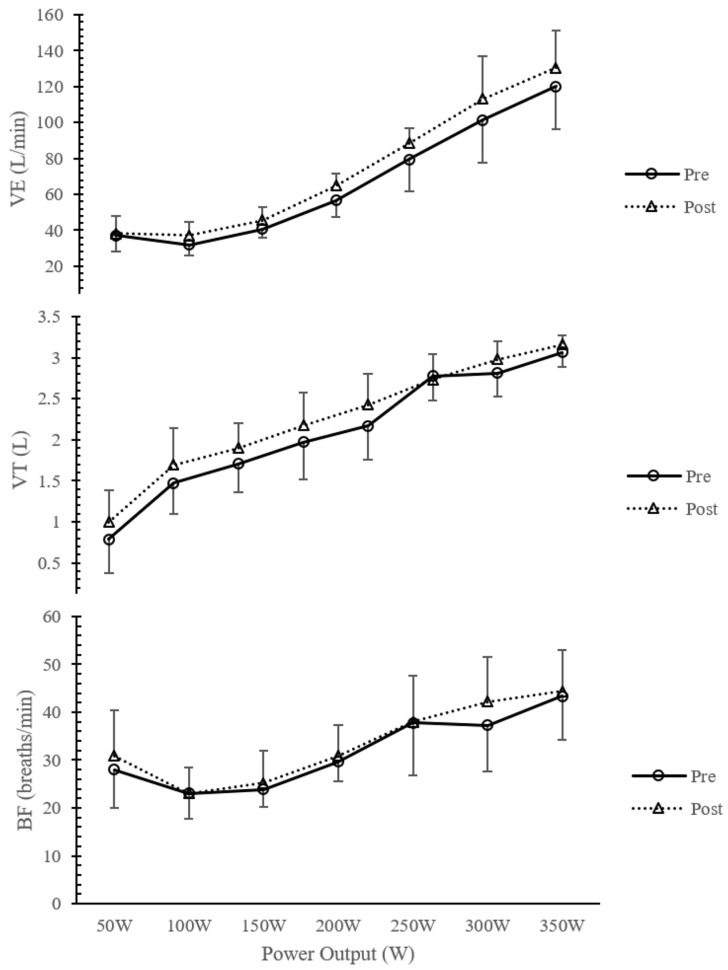
Breathing pattern analysis before (black) and after (dotted) IMT in the IMTG: ventilation (VE); tidal volume (VT); breathing frequency (BF).

**Figure 2:**
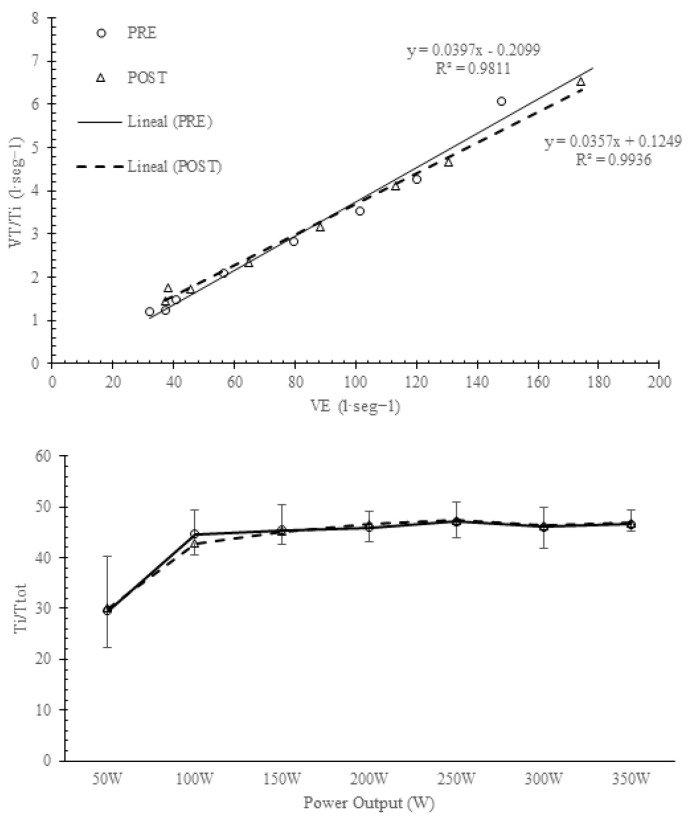
Evolution of driving impulse (VT/Ti) and timing (Ti/Ttot) before and after training in the IMTG.

**Figure 3:**
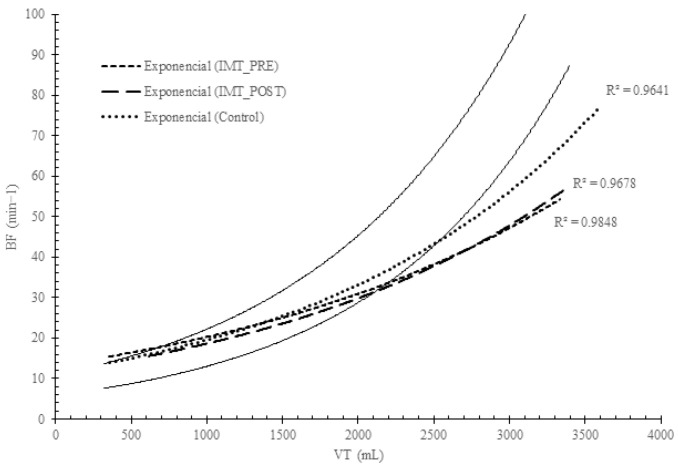
Breathing pattern analysis in the CON and IMTG before and after IMT based on Naranjo et al.’s nomogram [9].

Table 1 shows the analysis of spirometry variables and the Pimax before and after IMT. Pimax increased significantly in the IMTG (*p* < 0.05). Table 2 compares the breathing pattern responses during the incremental exercise test between groups before training. No significant differences were found before the intervention between groups (IMTGvsCON) in any variable analyzed in the study (*p* > 0.05, Table 2). Figure 1 describes the VE, VT, and BF responses before and after training in the IMTG. Figure 2 shows the evolution of VT/Ti (as VE increases) and Ti/Ttot (in relation to power output) before and after training in the IMTG. In Figure 3, we can see the relationship between VT and BF compared to the nomogram of [9] for the CON and IMTG, both before and after training.

## 4. Discussion

The main finding of this study was that inspiratory muscle training does not significantly modify the breathing pattern in healthy and active cyclists, despite producing an improvement in inspiratory muscle strength.

The traditional and commonly used method for evaluating breathing patterns in clinical applications is to analyze the VT and BF responses [2,19]. Hey et al. [19] observed that VT increased linearly during exercise until 50% of the FVC. Beyond this breakpoint, an increase in VE occurred due to changes in BF rather than VT. When this analysis was applied to healthy, trained, and untrained subjects during exercise, the relationship between VT and BF was found to be exponential, as it was described in the nomogram by [9]. In our study, we used this approach to analyze breathing patterns. Figure 3 illustrates the exponential response in the CON and IMTG before and after training. In both groups, the exponential curve closely approaches the lower limit of the nomogram proposed by [9], indicating a normal and low-energy breathing pattern response. Based on the position of both curves on the nomogram after IMT, we could consider that the IMT intervention did not significantly influence the breathing pattern. This lack of influence is likely due to the initially highly efficient breathing pattern observed in the IMTG before training. 

Milic-Emili and Grunstein [3] proposed a new analysis based on the central activity of a breathing pattern using the VT/Ti and Ti/Ttot relationships. Figure 1 and Figure 2 compare breathing patterns before and after training in the IMTG using this methodology. No differences were found at any intensity after training in the IMTG (Table 2). In our healthy subjects, the central regulation of the VE response to exercise intensity is primarily promoted by an increase in VT/Ti and a constant Ti/Ttot output (Figure 2), without changes in mechanical control (Figure 1). This similar behavior has been reported after endurance training [5,6] and is considered the normal response to exercise in healthy subjects. 

Regarding the influence of IMT on the breathing pattern, to our knowledge, only one study has investigated this relationship. Charususin et al. [20] evaluated the effect of IMT on the breathing pattern in patients with chronic obstructive pulmonary disease (COPD). In these patients, the breathing pattern improved after 8 weeks of IMT. COPD patients usually develop a rapid and shallow breathing pattern, which is energetically opposite to the pattern required to minimize the work of breathing [21]. This inefficient breathing pattern is related to the restriction of VT expansion and might also be related to an imbalance between the load and capacity relationship of the inspiratory muscles [20]. On the other hand, hypoxia could be a factor that might promote changes in the breathing pattern. Our group reported that ventilator efficiency improved after IMT only in hypoxic conditions (FiO_2_ = 16.45%) but not in normoxia [22]. Further analysis would be necessary to investigate the effect of hypoxia on the breathing pattern during exercise. 

The lack of changes in the breathing pattern in our subjects after IMT could be explained by the initial efficient breathing pattern reported before training (Figure 3). As it has been described before, subjects with respiratory diseases and inefficient breathing patterns can significantly modify their ventilatory response after IMT. Therefore, we could consider that when there is not a stressful factor such as respiratory disease or altitude, the nervous system regulates the breathing pattern independently of inspiratory muscle strength. 

The main limitation of this study could be the sample size. Although it was adequate for detecting changes in the Pimax within the intervention group, it might have been insufficient for identifying differences between the CON and IMTG after the intervention period (Table 1). In fact, when we applied Cohen’s d effect size analysis [23] for the Pimax between groups after training, it was moderate (d = 0.68), indicating that with a larger sample size, the change could have been significant. 

## 5. Conclusions

In conclusion, IMT was not an effective intervention for improving the breathing pattern response during an incremental exercise test in healthy athletes. Our results, along with the evidence reported previously, indicate that the breathing pattern response is not related to fitness level or respiratory muscle performance. Driving and timing analyses suggest that the nervous system adjusts ventilation to exercise intensity independently of respiratory muscle strength. Our results not only provide valuable insights into the impact of IMT on breathing patterns, but also offer practical implications for athletes and healthcare professionals involved in exercise prescription. The main practical application is that IMT is an effective and safe intervention that enhances exercise capacity without causing changes or disruptions in the respiratory pattern, making it potentially applicable to any population.

## Figures and Tables

**Table 1:** Spirometry variables measured before (pre) and after (post) the 6-week intervention period.

	IMTG		CON	
	Pre	Post	Pre	Post
VC (L)	5.53 ± 0.9	5.17 ± 1.14	5.24 ± 1.1	5.11 ± 1.06
FVC (L)	5.46 ± 1	4.8 ± 1.35	5.06 ± 1.1	4.96 ± 0.93
FEV1 (L)	4.64 ± 0.92	4.19 ± 0.80	4.31 ± 0.85	4.06 ± 0.79
FEV1/VC (%)	84.13 ± 11.58	82.51 ± 9.19	82.33 ± 6.28	79.84 ± 6.48
PEF (L·min^−1^)	9.27 ± 2.23	8.20 ± 1.53	8.90 ± 2.47	8.73 ± 2.40
PIF (L·min^−1^)	7.04 ± 1.92	8.31 ± 2.39	7.12 ± 1.20	6.73 ± 3.26
Pimax (cmH_2_O)	119.66 ± 37.36	166.91 ± 42.65 *	130.55 ± 33.58	130.42 ± 61.93

Data are presented as Mean ± Standard deviation. *p* indicates *p*-value. * significant differences (*p* < 0.05).

**Table 2:** Comparison between groups (IMTGvsCON) before and after the 6-week IMT.

*Pre-IMT*															
	*VE* (L·min^−1^)			*BF* (Breahts·min^−1^)			*VT* (L)			*VT/Ti* (L·seg)			*Ti/Ttot*		
	IMTG	CON	*p*	IMTG	CON	*p*	IMTG	CON	*p*	IMTG	CON	*p*	IMTG	CON	*p*
50 W	35.11 ± 13.97	33.89 ± 13.91	0.855	28 ± 7.98	30.11 ± 8.57	0.596	0.79 ± 0.41	0.77 ± 0.41	0.930	1.23 ± 0.36	1.26 ± 0.3	0.894	29.56 ± 10.6	30.11 ± 9.9	0.911
100 W	31.89 ± 6.11	30.11 ± 9.98	0.655	23 ± 5.24	23.89 ± 10.64	0.825	1.47 ± 0.38	1.62 ± 0.52	0.807	1.21 ± 0.47	1.09 ± 0.4	0.500	44.67 ± 4.6	47.11 ± 5.5	0.328
150 W	40.56 ± 4.42	41.22 ± 14.52	0.897	23.89 ± 3.69	25.78 ± 9.86	0.598	1.71 ± 0.34	1.91 ± 0.52	0.329	1.48 ± 0.42	1.64 ± 0.4	0.317	45.44 ± 5.0	44.67 ± 4.4	0.745
200 W	56.67 ± 9.3	56.33 ± 16.53	0.959	29.67 ± 4.12	31.11 ± 7.2	0.609	1.97 ± 0.45	2.03 ± 0.48	0.796	2.1 ± 0.49	2.2 ± 0.49	0.610	46 ± 3	45.33 ± 3.6	0.682
250 W	79.44 ± 17.76	83.33 ± 31.85	0.753	37.78 ± 10.88	37.11 ± 8.84	0.888	2.17 ± 0.41	2.43 ± 0.55	0.271	2.82 ± 0.88	3.08 ± 0.8	0.473	47.11 ± 3.8	47.33 ± 3.2	0.895
300 W	101.29 ± 23.44	107 ± 36.7	0.726	37.29 ± 9.62	44.89 ± 12.21	0.242	2.78 ± 0.3	2.42 ± 0.53	0.134	3.52 ± 1.06	4.01 ± 1.1	0.298	46.14 ± 3.8	45.89 ± 4	0.910
350 W	120.17 ± 24.1	124.6 ± 31.19	0.796	43.33 ± 9.14	49.6 ± 6.35	0.253	2.81 ± 0.28	2.56 ± 0.64	0.402	4.27 ± 1.2	4.44 ± 1.2	0.782	46.67 ± 2.6	47.8 ± 2.2	0.473
*Post-IMT*															
	*VE* (L·min^−1^)			*BF* (breahts·min^−1^)			*VT* (L)			*VT/Ti* (L·seg)			*Ti/Ttot*		
	IMTG	CON	*p*	IMTG	CON	*p*	IMTG	CON	*p*	IMTG	CON	*p*	IMTG	CON	*p*
50 W	38.11 ± 9.81	31.11 ± 6.15	0.089	30.88 ± 9.53	29.44 ± 4.30	0.684	1 ± 0.38	0.77 ± 0.16	0.135	1.73 ± 0.78	1.37 ± 0.3	0.209	30 ± 7.71	28.55 ± 7.98	0.701
100 W	37.22 ± 7.64	30.77 ± 6.49	0.072	23 ± 5.5	21.44 ± 4.63	0.526	1.69 ± 0.44	1.53 ± 0.37	0.424	1.45 ± 0.26	1.36 ± 0.5	0.667	42.77 ± 2.1	42 ± 10.07	0.824
150 W	45.66 ± 7.33	46.44 ± 7.76	0.830	25.22 ± 6.74	25.88 ± 6.33	0.832	1.9 ± 0.3	1.81 ± 0.35	0.574	1.72 ± 0.33	1.71 ± 0.2	0.970	45.22 ± 2.48	44.11 ± 4.75	0.543
200 W	64.88 ± 6.62	58.11 ± 8.23	0.072	30.88 ± 6.37	28.77 ± 6.33	0.491	2.17 ± 0.39	2.06 ± 0.44	0.587	2.33 ± 0.31	2.17 ± 0.3	0.301	46.66 ± 3.6	44.44 ± 5.19	0.308
250 W	88.33 ± 8.42	81.5 ± 13.52	0.224	38 ± 9.55	34.12 ± 8.11	0.385	2.25 ± 0.26	2.35 ± 0.52	0.741	3.15 ± 0.35	2.79 ± 0.7	0.200	46.75 ± 2.91	47.12 ± 6.72	0.902
300 W	113.25 ± 23.83	116.1 ± 5.82	0.771	42.25 ± 9.19	50.37 ± 10.41	0.120	2.8 ± 0.23	2.42 ± 0.48	0.111	4.1 ± 0.63	4.08 ± 0.6	0.973	45.16 ± 3.25	48.25 ± 2.31	0.307
350 W	130.5 ± 20.72	139.5 ± 11.38	0.456	44.33 ± 8.64	50.25 ± 12.76	0.187	3.01 ± 0.25	2.95 ± 0.64	0.917	4.66 ± 0.8	4.7 ± 0.6	0.929	47.25 ± 1.5	49.33 ± 3.78	0.180

Data are presented as Mean ± Standard deviation. *p* indicates *p*-value.

## Data Availability

Data is contained within the article.
